# Changes in volume and bouts of physical activity and sedentary time across early childhood: a longitudinal study

**DOI:** 10.1186/s12966-019-0805-6

**Published:** 2019-05-14

**Authors:** Jill A. Hnatiuk, Karen E. Lamb, Nicola D. Ridgers, Jo Salmon, Kylie D. Hesketh

**Affiliations:** 1 Institute for Physical Activity and Nutrition (IPAN), School of Exercise and Nutrition Sciences, Deakin University, Geelong, Australia; 2 Murdoch Children’s Research Institute, Royal Children’s Hospital, Parkville, Australia; 3 Department of Paediatrics, The University of Melbourne, Parkville, Australia

**Keywords:** Physical activity, Sedentary behaviour, Trajectories, Patterns, Bouts, Early childhood, Longitudinal, Accelerometry

## Abstract

**Background:**

Understanding changes in physical activity and sedentary time (SED) during early childhood may provide insights into how to effectively promote a healthy start to life. This study examined changes in total volume and bouts of SED, light- (LPA), and moderate- to vigorous-intensity physical activity (MVPA) across early childhood, and explored differences in change between boys and girls.

**Methods:**

Data were drawn from 330 children participating in the Melbourne InFANT Program, collected between 2008 and 2013 and analysed in 2017. Children’s physical activity and SED were assessed for at least 7 days at each timepoint using ActiGraph GT1M accelerometers at 19 months, 3.5 and 5 years of age. Total volume of SED (≤100 counts per minute [CPM]), LPA (101–1680 CPM) and MVPA (≥1681 CPM) were expressed as a percentage of wear time, and the frequency (number of bouts/day) and duration (mins/bout) of SED, LPA and MVPA bouts ≥1 min were calculated at each time point. Multilevel models with random intercepts and slopes were used to examine changes in total volume and bouts of SED, LPA and MVPA for boys and girls.

**Results:**

Compared to aged 19 months, children’s total volume of SED and LPA decreased at 3.5 and 5 years old, while MVPA increased. The frequency of SED bouts at 3.5 and 5 years was greater than at 19 months, but the duration was shorter. Additionally, the frequency and duration of LPA bouts was lower and MVPA bout frequency and duration was greater at 3.5 and 5 years. In general, there was no evidence of sex differences in trajectories of children’s physical activity and SED. However, variations in trajectory were observed at the individual child level.

**Conclusions:**

Children’s total volume and bouts of SED, LPA and MVPA change across early childhood, mostly in a favourable direction. Trajectories appear to be similar for boys and girls. Investigation of individual variation in trajectories is likely to provide greater insight into associations between physical activity and future health and behavioural outcomes.

## Background

Optimising physical activity and minimising sedentary time in early childhood (infancy – 5 years of age) is important for health [1]. Previous reviews have found that greater daily physical activity participation during early childhood is associated with fewer cardiovascular risk factors and lower adiposity, and improved cognitive development and bone density in early and later childhood [1]. Excessive time spent sedentary in early childhood, namely in screen-based behaviours, is negatively associated with these same health outcomes [2, 3]. Despite the growing trend towards promoting physical activity in early childhood, little is known about how children’s physical activity and sedentary time are accumulated, and how the total volume and patterns of accumulation (i.e., frequency and bouts) change over time. This is important for several reasons. Firstly, identifying how physical activity and sedentary time change across early childhood provides an indication of the age at which children may be most or least active, which can be used to inform the timing of intervention programming. Secondly, identifying typical patterns of accumulation of physical activity and sedentary time across early childhood can provide an indication of how physical activity initiatives might be structured at various ages to increase total volume of physical activity and minimise sedentary time, as well as optimise health outcomes for children.

Previous studies examining changes in total volume of daily physical activity and sedentary time have generally reported that physical activity declines and sedentary time increases from the age of 4–5 years onwards, likely coinciding with the commencement of formal schooling [4–6]. However, little information exists on changes in children’s total volume of physical activity and sedentary time prior to commencing primary school (i.e., from when children begin walking independently through to the preschool years). A recent longitudinal study from Switzerland that focused on children from 2.5 years of age found increased total physical activity and moderate-to-vigorous physical activity (MVPA) up to age six, with sedentary time remaining somewhat stable [7].

The majority of research on patterns of physical activity and sedentary time accumulation (i.e., bout frequency and duration) is drawn from cross-sectional samples of school-aged children and adolescents [8–11], rather than toddlers and pre-schoolers. Only a few studies have investigated bouts of sedentary time in preschool aged children [12–16]. Previous studies investigating relatively long bouts of sedentary time for this age group (i.e., ≥10 min) have shown contrasting findings. Two studies from Scandinavia (Norway and Sweden) investigated sedentary bouts > 10 min. One reported a total of 83 mins/day was spent in bouts of this duration [12], whilst the other reported over 300 min per day [14]. Studies from Australia and Canada that examined several different sedentary bout lengths (1–4 min; 5–9 min; ≥10 mins) in children reported that the greatest proportion of sedentary time was derived from bouts that were short in duration < 1 min [15] or 1–4 min in length [13, 16]. One of these studies [13] also examined changes in children’s sedentary time over the course of 1 year (from age 3–5 years to age 4–6 years). They found increases in the total time spent in 1–4 min bouts and in ≥10 min sedentary bouts, regardless of whether children had commenced primary school or not [13].

Despite emerging research on sedentary bouts in early childhood, we are not aware of any studies that have examined bouts of physical activity in children under five. Research on physical activity patterns of accumulation in school aged children and youth have found that 66% of 8–17 year old children’s physical activity was accumulated through sporadic (1–4 min) bouts of MVPA, 16% through short (5–9 min) bouts and 18% through long (> 10 min) bouts of MVPA [11]. Other research with 8–10 year old children has found that found most MVPA was accumulated in bouts less than 1 min and the mean duration of a LPA bout was just over 1 min [17]. However, patterns of accumulation can be heavily influenced by how data are collected, particularly when using accelerometry (such as through the use of short vs long epoch settings) [18], which may possibly explain some of the differences in bout duration observed between studies. Additionally, recent reviews have highlighted that there is a lack of consensus on how physical activity bouts are defined in the literature [19].

An additional consideration to changes in activity spectrum patterns in early childhood are the sex differences from a young age. Previous research has consistently shown that boys’ total volume of physical activity tends to be greater than girls [20]. There is also evidence that patterns of accumulation of sedentary time may differ by sex, with boys engaging in more very short (< 1 min) bouts of sitting than girls [15]. Exploring sex differences in physical activity and sedentary time from when children commence independent walking is important for providing insights into how to better design programming to optimise physical activity and minimise sedentary time for both sexes early in life.

In summary, limited evidence exists for how children’s total volume of physical activity and sedentary time change across early childhood. Furthermore, no studies have examined changes in physical activity and sedentary patterns in early childhood. Consequently, this study aims to address these gaps and examine changes in total volume and bouts of sedentary time, LPA and MVPA across early childhood, and to explore whether there are any differences between boys and girls.

## Methods

### Participants

Participants for this study were drawn from the Melbourne InFANT Program and Melbourne InFANT Program Follow-Up. The methods of this study have been previously described elsewhere [21–23]. Briefly, the Melbourne InFANT Program was a cluster-randomised controlled trial focused on childhood obesity prevention and delivered to first-time parents’ groups (existing groups made up of parents with children born around a similar time in their local area, run through government-funded maternal and child health centres in the state of Victoria, Australia) when children were 4–19 months of age. Children were then followed up at two later time points: 3.5 and 5 years old [23]. This study used data collected when children were 19 months (2009–2010), 3.5 (2011–2012) and 5 (2013) years of age. Of the original sample of 542 parent-child dyads recruited at baseline (child aged 4 months old), 480 were still enrolled in the study at intervention conclusion (child aged 19 months old), and 361 and 337 families consented to take part in the study when the children were aged 3.5 years and 5 years old, respectively. All mothers in the Melbourne InFANT Program and Melbourne InFANT Program Follow-Up gave written consent for themselves and their child to take part in the research. Ethics approval for this project was granted from the Deakin University Human Research Ethics Committee and the Victorian Government’s Office for Children.

### Physical activity and sedentary time

Children’s physical activity and sedentary time were objectively assessed at each time point using ActiGraph GT1M accelerometers (ActiGraph LLC, Pensacola, FL, USA). The accelerometers were worn on an elastic belt and placed over the child’s right hip, and parents were instructed to keep the accelerometer on the child during all waking hours for at least 7 days, removing only for sleeping and water-based activities. Data were collected in 15-s epochs.

Accelerometer data were processed using customised Excel macros. 10 min of consecutive zeros were considered to be non-wear time [13, 24]. Validated cut-points of ≤100 counts per minute (CPM), 101–1680 CPM, and ≥ 1680 CPM were applied to the data at all ages to distinguish sedentary time, LPA, and MVPA [25], respectively. The 70/80 rule was applied separately at each time point to determine a valid day (19 months = 7.4 h; 3.5 years = 6.6 h; 5 years = 6.9 h) [26]. The 70/80 rule represents non-missing counts for at least 80% of a standard measurement day, defined as the length of time that at least 70% of the sample wore the monitor [27, 28]. Children were included in the analyses for this study if they wore the accelerometer for at least 3 valid days [24, 27] during at least one time point, consistent with previous research [4]. Children’s total volume of sedentary time, LPA and MVPA, expressed as a percentage of wear time, was averaged across all valid days. The average number of bouts per day lasting ≥1 min (frequency) and the average duration in a bout (minutes/day) were also determined. Given there is presently no consensus on the ‘optimal’ bout length for young children [19], a one-minute bout length was selected based on previous research that has focused on children’s physical activity and sedentary time [11, 13] and the sporadic nature of young children’s physical activity [19]. No exceptions were allowed, which has been shown to increase time accumulated in longer bouts [18].

### Demographic information

Mothers reported demographic information about themselves and their child including: the sex of their child, their own education levels (low = secondary school or lower; medium = trade certificate/diploma; high = university degree or higher), and their child’s date of birth. The child’s date of birth enabled the calculation of the child’s decimal age, which was based on when the survey was completed. Mothers also reported whether their child was attending primary school at the 5 year old time point (yes/no).

### Data analysis

Data were analysed in 2017 using Stata v.14.0 [29]. A chi-squared test was used to examine whether the number of valid time points of data differed by maternal education. Analysis of variance (ANOVA) tests examined differences in total SED, LPA and MVPA for children who had commenced primary school at age five and those who had not. Linear multilevel models were used to examine changes in the following outcome variables over time: total volume of SED, LPA and MVPA expressed as a percentage of wear time; and frequency (bouts/day) and duration (mins/day) of bouts of SED, LPA, MVPA. Multilevel modelling was considered the most appropriate analysis technique as it can manage nested data and is robust for dealing with missing data, assuming the data are missing at random [30]. Each model included a random intercept for both child and parent group of recruitment. In addition, the coefficient of time was allowed to vary randomly by child to allow each child to have his or her own trajectory of change. To determine whether there was evidence that there was random variation in the trajectory of change across participants, likelihood ratio tests were used to compare multilevel models with and without a random slope for time (i.e., random intercept only vs. random slope and intercept). In addition, models with an unstructured covariance, which allowed for correlations between the random intercept and slope, were considered. For most outcome variables (total volume of SED, LPA, MVPA, SED and MVPA bout frequency, and SED bout duration) the random slope and intercept with unstructured covariance provided the best fit for the data. For the remaining outcome variables (LPA bout frequency, and LPA and MVPA bout duration), a random intercept was sufficient. Model residuals were checked for normality and constant variance using QQ-plots and plots of residuals against fitted values. All assumptions appeared reasonable.

Since participants of the Melbourne InFANT Program were recruited as part of a cluster randomised controlled trial, models were initially fitted to examine whether change in the outcomes differed by treatment group (i.e., intervention vs. control). However, there was no evidence of a group by time interaction for any of the outcome variables. Therefore, data were pooled from both groups for the remaining analyses to maximise the sample size.

An interaction between time and sex of the child was included in models to determine if trajectories of physical activity and sedentary time across early childhood differed by sex. All analyses controlled for intervention group and maternal education (as a proxy for socioeconomic status) at baseline. The analyses examining the frequency and duration of bouts also controlled for accelerometer wear time, given the outcome was expressed in bouts or minutes per day, rather than as a percentage of wear time. As time was included as a categorical predictor in analyses, post-hoc pairwise comparisons were conducted to examine the differences in average volume and bouts of SED, LPA and MVPA between all of the time points. The Bonferroni correction (set at *p* < 0.01) was used in post-hoc testing to adjust for the multiple comparisons made.

## Results

From the 492 children enrolled in the Melbourne InFANT Program at 19 months, 330 children (67%) had sufficient accelerometer data for at least one time point during the follow-up period (e.g., 19 months, 3.5 and/or 5 years), resulting in 681 data points for the analyses. A total of 141 children had one time point of valid data, and 189 had two or more time points of valid data. From the total sample (*n* = 330), 52.6% of children were male, and 59.2% of mothers had a university degree or higher, with the remainder having a trade certificate (24.0%) or secondary school or lower (16.8%). Mothers with a university degree or higher were more likely to have children with 2 time points of data (χ^2^ = 7.76, *p* < 0.05). Twelve percent of children in the sample were attending primary school at the 5 year old time point, however, there was no evidence that their total volume of SED, LPA or MVPA differed from those who were not attending school. Consequently, these data were pooled. Table 1 outlines the descriptive physical activity and SED results at each time point. Mean accelerometer wear time was 587.21 (69.38) mins/day at 19 months old, 626.92 (63.08) mins/day at 3.5 years old and 650.18 (61.93) mins/day at 5 years old.

**Table 1:** Mean (SD) total volume and bouts of SED, LPA & MVPA at 19 months, 3.5 years and 5 years of age^a,b,c^

	19 months			3.5 years			5 years		
	Boys (*n* = 160)	Girls (*n* = 144)	Combined (*n* = 304)	Boys (*n* = 71)	Girls (*n* = 86)	Combined (*n* = 157)	Boys (*n* = 80)	Girls (*n* = 81)	Combined (*n* = 161)
Total volume SED (% wear time/day)	52.1 (6.1)	53.7 (6.8)^d^	52.8 (6.5)	49.7 (6.0)	50.9 (5.7)	50.3 (5.9)	49.3 (6.5)	51.3 (6.1)^d^	50.3 (6.4)
Total volume LPA (% wear time/day)	39.6 (4.9)	38.5 (5.1)	39.1 (5.1)	38.9 (4.6)	38.3 (3.6)	38.6 (4.1)	3.75 (4.5)	36.6 (4.1)	37.0 (4.3)
Total volume MVPA (% wear time/day)	8.3 (2.6)	7.8 (2.8)	8.1 (2.7)	11.4 (3.4)	10.7 (3.6)	11.0 (3.5)	13.1 (3.8)	12.1 (3.2)	12.6 (3.5)
SED bout frequency (bouts/day)	72.1 (14.1)	77.0 (16.0)	74.5 (15.2)	83.2 (15.0)	84.8 (12.9)	84.1 (13.9)	87.5 (14.5)	89.9 (11.6)	88.7 (13.1)
LPA bout frequency (bouts/day)	78.6 (14.8)	77.1 (15.8)	77.9 (15.3)	81.9 (15.0)	77.1 (11.4)^d^	79.3 (13.3)	77.9 (15.6)	73.3 (12.7)^d^	75.6 (14.4)
MVPA bout frequency (bouts/day)	7.9 (4.2)	7.4 (4.1)	7.6 (4.2)	15.8 (6.9)	13.7 (6.9)	14.6 (7.0)	20.8 (8.1)	17.6 (7.1)^d^	19.2 (7.8)
SED bout duration (mins/bout)	3.2 (0.5)	3.2 (0.5)	3.3 (0.5)	2.9 (0.4)	2.8 (0.3)	2.9 (0.3)	2.8 (0.4)	2.8 (0.7)	2.8 (0.6)
LPA bout duration (mins/bout)	1.6 (0.1)	1.6 (0.1)^d^	1.6 (0.1)	1.6 (0.9)	1.5 (0.8)	1.5 (0.1)	1.5 (0.9)	1.5 (0.6)^d^	1.5 (0.1)
MVPA bout duration (mins/bout)	1.5 (0.2)	1.5 (0.3)	1.5 (0.2)	1.6 (0.2)	1.5 (0.2)	1.6 (0.2)	1.6 (0.2)	1.6 (0.2)	1.6 (0.2)

^a^*SED* sedentary time, *LPA* light-intensity physical activity, *MVPA* moderate- to vigorous-intensity physical activity

^b^Bouts defined in this study as any sedentary time, LPA or MVPA > 1 min

^c^All values are Mean (SD)

^d^Significant difference between boys and girls at *p* < 0.05

Compared to when children were aged 19 months, the mean percentage of time (total volume) spent in SED and LPA decreased at 3.5 (SED: β [95%CI] = − 1.60% [− 3.13, − 0.07]; LPA: β [95%CI] = − 1.40% [− 2.53, − 0.27]) and 5 years old (SED: β [95%CI] = − 2.32% [− 3.99, − 0.64]; LPA: β [95%CI] = − 2.66% [− 3.90, − 1.41]), while the percentage of time spent in MVPA increased at 3.5 and 5 years old (3.5 years: β [95%CI] = 3.04% [2.28, 3.80]; 5 years: β [95%CI] = 5.01% [4.19, 5.83]). Post-hoc tests revealed no differences in the percentage of SED and LPA between 3.5 and 5 years; however, MVPA significantly increased during this time (*d* [95%CI] =1.74% [1.01, 2.47]). No difference in trajectories of SED, LPA or MVPA were observed between boys and girls, though girls engaged in a greater percentage of sedentary time overall than boys (β [95%CI] = 1.50% [0.06, 2.95]).

Compared to when children were aged 19 months, the average frequency of bouts of SED was greater at 3.5 (SED: β [95%CI] = 3.47 [1.12, 5.81]) and 5 (SED: β [95%CI] = 4.31 [1.54, 7.07]) years of age, but the duration was shorter (3.5 years SED: β [95% CI] = − 0.30 min [− 0.42, − 0.17]; 5 years SED: β [95%CI] = − 0.35 min [− 0.52, − 0.18]). There was no evidence of a difference in either average SED bout duration or frequency between 3.5 and 5 years.

For LPA, compared to when children were aged 19 months, at 3.5 and 5 years the frequency (3.5 years: β [95%CI] = − 4.82 [− 8.27, − 1.38]; 5 years β [95%CI] = − 10.39 [− 13.77, − 7.00]) and duration (3.5 years: β [95%CI] = − 0.06 min [− 0.09, − 0.03]; 5 years β [95%CI] = − 0.11 min [− 0.14, − 0.08]) of LPA bouts decreased. There was also a decrease in LPA bout frequency (*d* [95%CI] = − 5.72 [− 8.96, − 2.49]) and duration (*d* [95%CI] = − 0.06 min [− 0.09, − 0.03]) between 3.5 and 5 years.

For MVPA, compared to child aged 19 months, average MVPA bout frequency was greater at 3.5 years (β [95%CI] = 6.96 [5.57, 8.35]) and 5 years (β [95%CI] = 12.31 [10.61, 14.01]), as was MVPA bout duration (3.5 years: β [95%CI] = 0.11 min [0.05, 0.17]; 5 years: β [95%CI] = 0.21 min [0.07, 0.18]). Between 3.5 and 5 years there was an increase in MVPA bout frequency (*d* [95%CI] = 4.63 [3.28, 5.98]), but not duration. Girls engaged in more sedentary bouts (β [95%CI] = 2.82 [0.28, 5.36]) and shorter LPA bouts (β [95%CI] = − 0.25 [− 0.05, − 0.001]) overall than boys (main effects) and their trajectory of MVPA bout frequency was less steep at 5 years of age (β [95%CI] = − 2.44 [− 4.84,-0.04]) (sex X time interaction). No further sex differences were observed for any of the other variables examined (*p*-values all > 0.05).

**Fig. 1:**
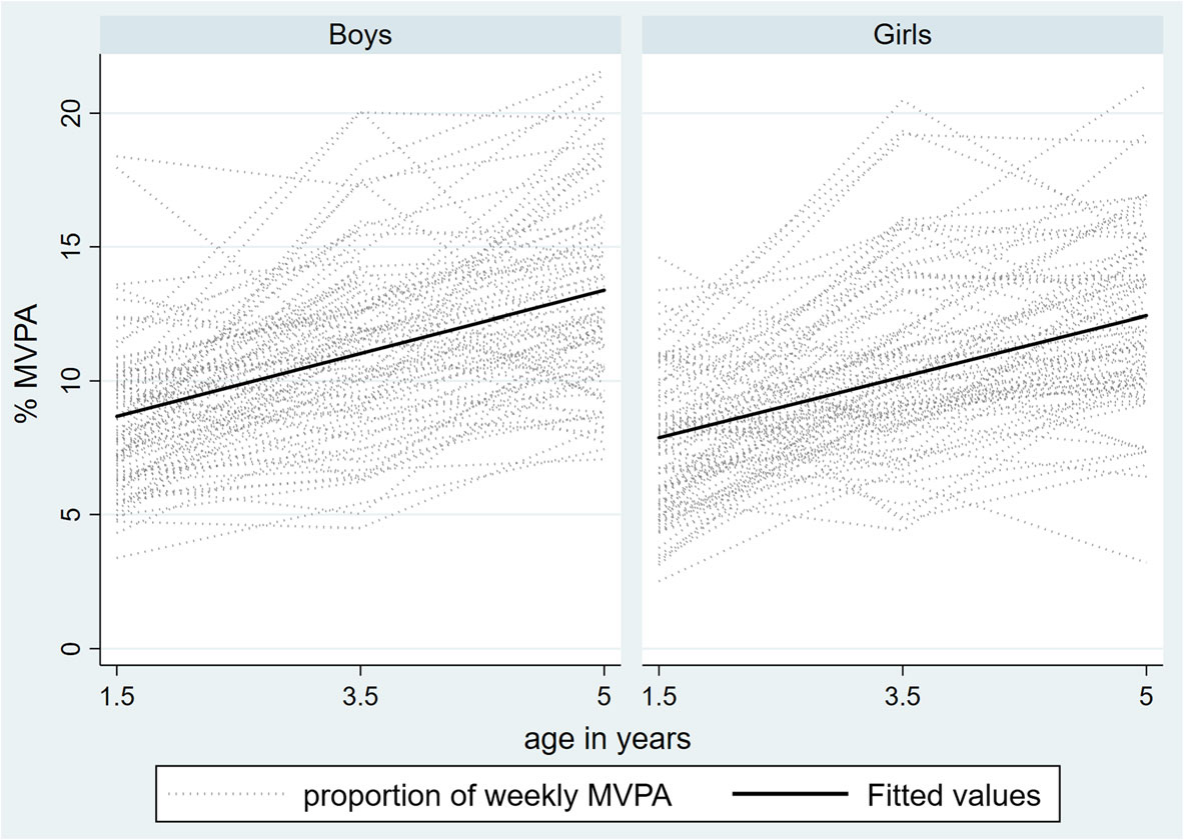
Boys’ and girls’ moderate- to vigorous-intensity physical activity (MVPA) by age, highlighting varied trajectories across early childhood. Fitted values attained from multilevel models examining change in children’s average daily percentage of MVPA. Models controlled for intervention group and maternal education

Table 2 reports the estimated standard deviations and confidence intervals for the random effects (random intercept for child and parent group, random slope for time) from the models of total volume of SED, LPA and MVPA. For these outcome variables, there was evidence of variability in the coefficient of time between children. This means that the slopes (trajectories) of SED, LPA and MVPA varied between individual children (see Fig. 1 for sample depiction of individual trajectories, separated by sex). The findings shown in Table 2 also highlight that there was a negligible amount of variability in SED, LPA and MVPA between parent intervention groups.

**Table 2:** Random effects parameters for total volume of SED, LPA and MVPA^a,b^

Variable	Random effects parameter	Standard deviation (95% CI)
Total volume SED (% wear time/day)	Parents group attended	2.98^− 7^ (2.57^− 16^, 346.35)
	Child	9.18 (1.42, 59.10)
	Time	2.49 (0.48, 12.96)
	Residual	5.31 (4.18, 6.75)
Total volume LPA (% wear time/day)	Parents group attended	4.35^−8^ (0.00, 0.00)
	Child	8.28 (5.72, 11.98)
	Time	2.03 (1.38, 2.98)
	Residual	3.93 (3.50, 4.41)
Total volume MVPA (% wear time/day)	Parents group attended	2.05^−7^ (1.19^−10^, 3.52^−4^)
	Child	2.34 (0.41, 13.33)
	Time	1.04 (0.57, 1.90)
	Residual	2.67 (2.34, 2.97)

^a^*SED* sedentary time, *LPA* light-intensity physical activity, *MVPA* moderate- to vigorous-intensity physical activity

^b^Similar estimates of associations observed when parent group attended was omitted from the models. Variable was retained in model given the study design

## Discussion

This study was the first to examine changes in total daily volume and bouts of SED, LPA and MVPA across early childhood between boys and girls, beginning from around the time when children commenced independent walking. Overall, generally favourable trends were observed across the sample. As children aged, a greater proportion of time was spent in MVPA, which appeared to be replacing some of the time spent sedentary and in LPA. This suggests that the recommendation to gradually increase physical activity intensity during the early childhood period (i.e., 60 mins MVPA of energetic play from 3 to 5 years of age [31, 32]) is consistent with the trajectory of children’s objectively assessed MVPA, making it a feasible recommendation for this age group. Given the favourable effects of MVPA on children’s cardiovascular health [33] the progressive replacement of SED and LPA with MVPA is a promising finding. The progressive increase could be due to favourable environmental changes (e.g., less time in restricted movement; greater opportunities for engagement in MVPA with other children or family members, more independence) and/or improved motor development (i.e., from intermittent walking to more sustained activity) at these later ages [34].

Since the mean duration of MVPA bouts increased only slightly over time, our findings suggest that the higher total volume of MVPA occurring at 3.5 and 5 years resulted from progressively more bouts rather than from more prolonged MVPA. By 5 years of age almost 1/3 of children’s MVPA was accumulated through bouts > 1 min, compared to about 1/5 at 19 months old. This is possibly due to changes in children’s growth and development [35], perhaps coinciding with greater opportunities for active play and less time in situations restricting movement [36].

Little research has investigated physical activity bouts in children, with none in the early childhood period. Previous research on primary school-aged children has reported mixed results regarding the typical bout duration for that age group. One study found that the average bout duration in a sample of 12 year old children was 4.7 min, representing approximately 40% of their total MVPA time [37]. Others conducted with 6–10 year old children [17, 38] and 14-year old adolescent boys [39] found most MVPA was accumulated in bouts less than 1 min; for example, Sanders at et., [39] found an average bout duration of just 4.1 s. Given that previous research has shown that methodological decisions, in particular the epoch length selected when using accelerometers, can heavily influence the results attained [18], it is likely some of the differences in bout duration estimates are a result of this. Nonetheless, our study findings suggest that activities that are quite brief in duration (< 1 min) still enable young children to accumulate sufficient volumes of MVPA throughout the day. Given this, interventions in this age group may want to focus on providing opportunities to increase the number of MVPA bouts occurring throughout the day. For example, early learning centres might increase the frequency of outdoor play periods during care (as has been tested previously [40]), or parents might be supported to encourage play activities that facilitate spurts of MVPA (e.g., chasing games) throughout the day at home.

A decrease in the duration of sedentary bouts between 19 months and 3.5 and 5 years old, but an increase in the frequency of these bouts, was also observed. However, no differences in total volume or bout frequency and duration were observed between 3.5 and 5 year time points. This suggests that the biggest change in how sedentary time is accumulated occurs between 19 months and 3.5 years, with minimal change thereafter. It is difficult to postulate why this finding has occurred, but could be due to the combined effect of developmental and environmental changes. For example, the increasingly social play with other children that occurs between 19 months and 3.5 years (shifting from solitary/parallel play to associative or cooperative play [41]) may impact on sedentary bout frequency, bout duration and total volume of sedentary time. Alternatively, changing parent or caregiver actions that might occur to a greater degree between 18 months and 3.5 years (compared to 3.5 and 5 years old), such as less reliance on a pram, may reduce the duration of sedentary bouts and total volume of sedentary time. However, as these are just hypotheses, there appears to be important research opportunities for understanding sedentary behaviour in this very young (< 2 year old) sample group given that their sedentary time appears to be accumulated differently to that in the preschool years. A noticeable increase in sedentary bout length may not appear again until children transition to school, at which point the structure and policies within the present school environment may negatively impact children’s total volume of sedentary time [13]. It is important to note that within our sample, despite more frequent sedentary bouts ≥1 min occurring over time, these were not enough to increase children’s total volume of sedentary time. At present the relationship between frequency and duration of bouts of sedentary time and children’s health is not known, so the overall reduction in total volume of sedentary time between 19 months and 3.5 and 5 years appears to be a favourable outcome, irrespective of how sedentary time was accumulated.

Evidence of some sex differences in both volume and bouts of physical activity and sedentary time were also noted, though these were predominantly main effects rather than interactions over time (i.e., changes in trajectory). This supports previous work that has suggested that girls engage in more sedentary time and less MVPA than boys [20, 42], but also proposes that the trajectories of physical activity and sedentary behaviour between the sexes are not dissimilar. The only exception was that whilst MVPA bout frequency increased from 19 months among girls and boys, girls appeared to increase to a lesser extent than boys by 5 years old, although the effect size of this difference was small (approximately two fewer bouts/day). Nonetheless, it may be important for care providers and parents to pay particular attention to prompting girls to engage in MVPA more regularly across early childhood to minimise the sex differences already apparent in MVPA during this period [20] and continuing later in life [43]. Although there is limited evidence on strategies to support girls’ physical activity participation in the early years, some ideas can be drawn from the DADEE program, a father-daughter physical activity intervention conducted with primary school girls [44]. In this program, the authors highlight the importance of program aspects such as redefining gender norms, participating in co-physical activity and improving fundamental movement skills for successfully increasing physical activity amongst girls [44]. Perhaps the efficacy of a similar approach, extended to a range of care providers, could be investigated in early childhood, especially during the preschool years where the sex difference in MVPA bout frequency seems to emerge.

A novel finding of this work was that whilst the total volume of MVPA increased and LPA and sedentary behaviour decreased over time, individual children’s activity trajectories, particularly with respect to children’s MVPA, differed. Visual inspection of the data showed that some children demonstrated a linear increase or decrease over time, whilst others showed a ‘V’, or inverted ‘V’ shape, over time. Understanding how these different trajectories of physical activity and sedentary behaviour in early life are associated with future physical activity and sedentary behaviour patterns, as well as with health outcomes, will be important for future research in this field. Additionally, identifying the modifiable factors that predict different trajectories of physical activity and sedentary behaviour across early childhood may be useful for informing intervention programs. For example, early targeted support could be provided to those children who demonstrate decreasing physical activity and increased sedentary patterns.

### Strengths and limitations

The strengths of this work include the objective measurement of physical activity and sedentary time, the inclusion of both volume and bouts of different intensities of physical activity and sedentary time, as well as multiple time points across the early childhood period. Nonetheless, we recognise that this study is not without its limitations. It is possible that some change in bout frequency or duration may have occurred over this time, but were less than 1 min in length and therefore not taken into account with the present analysis strategy. However, it was decided a priori that the bout duration of greater than 1 min would be used for both physical activity and sedentary time, consistent with previous research [13, 15] to enable comparisons and the brief nature of young children’s physical activity [38]. This is particularly relevant given that no consensus on ‘long’ vs. ‘short’ duration bouts have been established at present [19]. Additionally, some of the variability in physical activity and sedentary time estimates at each time point could be due to measurement error, potentially impacting the trajectories identified. There is also currently considerable challenges with the application of cut-points for children during the early childhood years [45]. This study used the same cut-points across each time point to ensure that any changes in physical activity detected were a result of changed movement behaviour rather than a change in cut-point. However, as some evidence suggests that cut-points should change with age [46], it is possible that the application of the same cut-point across these three time periods may have misclassified some movement behaviours. Lastly, attrition occurred over time, a proportion of the sample did not have all three time points of data and the use of 15-s epochs may underestimated sedentary time and MVPA, and overestimated LPA.

## Conclusions

This is the first study to focus on changes in young children’s volume and bouts of physical activity and sedentary time across the early childhood period. This study found that from 19 months, children’s MVPA increased, whilst their LPA and sedentary time decreased, largely a result of changes in bout frequency. Trajectories of physical activity were mostly similar for boys and girls, but showed different patterns between individual children in the sample. Future research should consider examining the implications of different trajectories of physical activity and sedentary time across early childhood on children’s later physical activity and sedentary behaviours, as well as associations with health.

## Abbreviations

CPM: Counts per minute
LPA: light-intensity physical activity
MVPA: Moderate- to vigorous-intensity physical activity
SED: Sedentary time

## References

[1] Carson V, Lee E-Y, Hewitt L, Jennings C, Hunter S, Kuzik N (2017). Systematic review of the relationships between physical activity and health indicators in the early years (0-4 years). BMC Public Health.

[2] LeBlanc AG, Spence JC, Carson V, Gorber SC, Dillman C, Janssen I (2012). Systematic review of sedentary behaviour and health indicators in the early years (aged 0-4 years). Appl Physiol Nutr Metab.

[3] Poitras VJ, Gray CE, Janssen X, Aubert S, Carson V, Faulkner G (2017). Systematic review of the relationships between sedentary behaviour and health indicators in the early years (0–4 years). BMC Public Health.

[4] Cooper AR, Goodman A, Page AS, Sherar LB, Esliger DW, van Sluijs EM (2015). Objectively measured physical activity and sedentary time in youth: the international children’s accelerometry database (ICAD). Int J Behav Nutr Phys Act.

[5] Taylor RW, Murdoch L, Carter P, Gerrard DF, Williams SM, Taylor BJ (2009). Longitudinal study of physical activity and inactivity in preschoolers: the FLAME study. Med Sci Sports Exerc.

[6] Taylor RW, Williams SM, Farmer VL, Taylor BJ (2013). Changes in physical activity over time in Young children: a longitudinal study using accelerometers. PLoS One.

[7] Schmutz EA, Haile SR, Leeger-Aschmann CS, Kakebeeke TH, Zysset AE, Messerli-Bürgy N (2018). Physical activity and sedentary behavior in preschoolers: a longitudinal assessment of trajectories and determinants. Int J Behav Nutr Phys Act.

[8] Saunders TJ, Tremblay MS, Mathieu M-È, Henderson M, O’Loughlin J, Tremblay A (2013). Associations of sedentary behavior, sedentary bouts and breaks in sedentary time with cardiometabolic risk in children with a family history of obesity. PLoS One.

[9] Júdice PB, Silva AM, Berria J, Petroski EL, Ekelund U, Sardinha LB (2017). Sedentary patterns, physical activity and health-related physical fitness in youth: a cross-sectional study. Int J Behav Nutr Phys Act.

[10] Gabel L, Ridgers N, Della Gatta P, Arundell L, Cerin E, Robinson S (2016). Associations of sedentary time patterns and TV viewing time with inflammatory and endothelial function biomarkers in children. Pediatr Obes.

[11] Mark AE, Janssen I (2009). Influence of bouts of physical activity on overweight in youth. Am J Prev Med.

[12] Andersen Eivind, Borch-Jenssen Janne, Øvreås Steinar, Ellingsen Hanna, Jørgensen Kari Anne, Moser Thomas (2017). Objectively measured physical activity level and sedentary behavior in Norwegian children during a week in preschool. Preventive Medicine Reports.

[13] Carson V, Salmon J, Crawford D, Hinkley T, Hesketh KD (2016). Longitudinal levels and bouts of objectively measured sedentary time among young Australian children in the HAPPY study. J Sci Med Sport.

[14] Berglind D, Tynelius P (2017). Objectively measured physical activity patterns, sedentary time and parent-reported screen-time across the day in four-year-old Swedish children. BMC Public Health.

[15] Ellis YG, Cliff DP, Janssen X, Jones RA, Reilly JJ, Okely AD (2017). Sedentary time, physical activity and compliance with IOM recommendations in young children at childcare. Prev Med Rep.

[16] Kuzik N, Clark D, Ogden N, Harber V, Carson V (2015). Physical activity and sedentary behaviour of toddlers and preschoolers in child care centres in Alberta, Canada. Can J Public Health.

[17] Baquet G, Stratton G, Van Praagh E, Berthoin S (2007). Improving physical activity assessment in prepubertal children with high-frequency accelerometry monitoring: a methodological issue. Prev Med.

[18] Aadland E, Andersen LB, Anderssen SA, Resaland GK, Kvalheim OM (2018). Associations of volumes and patterns of physical activity with metabolic health in children: a multivariate pattern analysis approach. Prev Med.

[19] Verswijveren SJ, Lamb KE, Bell LA, Timperio A, Salmon J, Ridgers ND (2018). Associations between activity patterns and cardio-metabolic risk factors in children and adolescents: a systematic review. PLoS One.

[20] Bingham DD, Costa S, Hinkley T, Shire KA, Clemes SA, Barber SE (2016). Physical activity during the early years: A systematic review of correlates and determinants. Am J Prev Med.

[21] Campbell K. J., Lioret S., McNaughton S. A., Crawford D. A., Salmon J., Ball K., McCallum Z., Gerner B. E., Spence A. C., Cameron A. J., Hnatiuk J. A., Ukoumunne O. C., Gold L., Abbott G., Hesketh K. D. (2013). A Parent-Focused Intervention to Reduce Infant Obesity Risk Behaviors: A Randomized Trial. PEDIATRICS.

[22] Campbell KJ, Hesketh K, Crawford D, Salmon J, Ball K, McCallum Z (2008). The Infant feeding activity and nutrition trial (INFANT) an early intervention to prevent childhood obesity: cluster-randomised controlled trial. BMC Public Health.

[23] Hesketh KD, Campbell K, Salmon J, McNaughton SA, McCallum Z, Cameron A (2013). The Melbourne Infant feeding, activity and nutrition trial (InFANT) program follow-up. Contemp Clin Trials.

[24] Hinkley T, O'Connell E, Okely AD, Crawford D, Hesketh K, Salmon J (2012). Assessing volume of accelerometry data for reliability in preschool children. Med Sci Sports Exerc.

[25] Pate RR, Almeida MJ, McIver KL, Pfeiffer KA, Dowda M (2006). Validation and calibration of an accelerometer in preschool children. Obesity.

[26] Ridgers ND, Timperio A, Crawford D, Salmon J (2012). Five-year changes in school recess and lunchtime and the contribution to children's daily physical activity. Br J Sports Med.

[27] Hnatiuk J, Ridgers ND, Salmon J, Campbell K, McCallum Z, Hesketh K (2012). Physical activity levels and patterns of 19-month-old children. Med Sci Sports Exerc.

[28] Catellier DJ, Hannan PJ, Murray DM, Addy C, Conway T, Yang S (2005). Imputation of missing data when measuring physical activity by accelerometry. Med Sci Sports Exerc.

[29] StataCorp. Stata Statistical Software: Release 14. College Station: StataCorp LP; 2015.

[30] Twisk J (2006). Applied multilevel analysis.

[31] Department of Health (2017). Australian 24-hour movement guidelines for the early years (birth to 5 years): an integration of physical activity, sedentary behaviour and sleep.

[32] Tremblay MS, Chaput J-P, Adamo KB, Aubert S, Barnes JD, Choquette L (2017). Canadian 24-hour movement guidelines for the early years (0–4 years): an integration of physical activity, sedentary behaviour, and sleep. BMC Public Health.

[33] Poitras VJ, Gray CE, Borghese MM, Carson V, Chaput J-P, Janssen I (2016). Systematic review of the relationships between objectively measured physical activity and health indicators in school-aged children and youth 1. Appl Physiol Nutr Metab.

[34] Haywood KMG, Nancy (2009). Lifespan motor development.

[35] Malina RM, Bouchard C, Bar-Or O (2004). Heart, blood and lungs. Growth, maturation and physical activity.

[36] Hesketh KD, Crawford DA, Abbott G, Campbell KJ, Salmon J (2015). Prevalence and stability of active play, restricted movement and television viewing in infants. Early Child Dev Care.

[37] Brooke HL, Atkin AJ, Corder K, Brage S, van Sluijs EMF (2016). Frequency and duration of physical activity bouts in school-aged children: a comparison within and between days. Prev Med Rep.

[38] Bailey RC, Olson J, Pepper SL, Porszasz J, Barstow TJ, Cooper DM (1995). The level and tempo of children’s physical activities: an observational study. Med Sci Sports Exerc.

[39] Sanders T, Cliff DP, Lonsdale C (2014). Measuring adolescent Boys' physical activity: bout length and the influence of accelerometer epoch length. PLoS One.

[40] Razak LA, Yoong SL, Wiggers J, Morgan PJ, Jones J, Finch M (2018). Impact of scheduling multiple outdoor free-play periods in childcare on child moderate-to-vigorous physical activity: a cluster randomised trial. Int J Behav Nutr Phys Act.

[41] Sigelman CR, Elizabeth (2012). Lifespan human development.

[42] Byun W, Dowda M, Pate RR (2011). Correlates of objectively measured sedentary behavior in US preschool children. Pediatrics.

[43] Sterdt E, Liersch S, Walter U (2014). Correlates of physical activity of children and adolescents: a systematic review of reviews. Health Educ J.

[44] Morgan Philip J, Young Myles D, Barnes Alyce T, Eather Narelle, Pollock Emma R, Lubans David R (2018). Engaging Fathers to Increase Physical Activity in Girls: The “Dads And Daughters Exercising and Empowered” (DADEE) Randomized Controlled Trial. Annals of Behavioral Medicine.

[45] Hnatiuk J, Salmon J, Hinkley T, Okely A, Trost SG (2014). A review of preschool children's physical activity and sedentary time using objective measures. Am J Prev Med.

[46] Sirard JR, Trost SG, Pfeiffer KA, Dowda M, Pate RR (2005). Calibration and evaluation of an objective measure of physical activity in preschool children. J Phys Act Health.

